# Metabolic Signatures Associated with Severity in Hospitalized COVID-19 Patients

**DOI:** 10.3390/ijms22094794

**Published:** 2021-04-30

**Authors:** Judith Marín-Corral, Jose Rodríguez-Morató, Alex Gomez-Gomez, Sergi Pascual-Guardia, Rosana Muñoz-Bermúdez, Anna Salazar-Degracia, Purificación Pérez-Terán, Marcos I. Restrepo, Olha Khymenets, Noemí Haro, Joan Ramon Masclans, Oscar J. Pozo

**Affiliations:** 1 Critical Care Department, Hospital del Mar, 08003 Barcelona, Spain; jmarincorral@gmail.com (J.M.-C.); mbrosana@gmail.com (R.M.-B.); asalazardegracia@gmail.com (A.S.-D.); puriteran@gmail.com (P.P.-T.); 2 Critical Illness Research Group (GREPAC), Institut Hospital del Mar d’Investigacions Mèdiques (IMIM), 08003 Barcelona, Spain; 3 Division of Pulmonary Diseases & Critical Care Medicine, University of Texas Health San Antonio, San Antonio, TX 78229, USA; spascualguardia@gmail.com (S.P.-G.); RESTREPOM@uthscsa.edu (M.I.R.); 4 Integrative Pharmacology and Systems Neuroscience Research Group, Neurosciences Research Program, IMIM-Institut Hospital del Mar d’Investigacions Mèdiques, 08003 Barcelona, Spain; jose.rodriguez@upf.edu (J.R.-M.); agomez@imim.es (A.G.-G.); okhymenets@imim.es (O.K.); nharo@imim.es (N.H.); 5 Department of Experimental and Health Sciences, Universitat Pompeu Fabra (CEXS-UPF), 08003 Barcelona, Spain; 6 Spanish Biomedical Research Centre in Physiopathology of Obesity and Nutrition (CIBEROBN), Instituto de Salud Carlos III (ISCIII), 28029 Madrid, Spain; 7 Respiratory Department, Hospital del Mar, IMIM, 08003 Barcelona, Spain; 8 CIBER de Enfermedades Respiratorias, 28029 Madrid, Spain; 9 Section of Pulmonary & Critical Care Medicine, South Texas Veterans Health Care System, San Antonio, TX 78229, USA; 10 Department of Medicine, Universitat Autònoma de Barcelona (UAB), 08193 Barcelona, Spain

**Keywords:** COVID-19, metabolomics, severity, kynurenine, ceramides

## Abstract

The clinical evolution of COVID-19 pneumonia is poorly understood. Identifying the metabolic pathways that are altered early with viral infection and their association with disease severity is crucial to understand COVID-19 pathophysiology, and guide clinical decisions. This study aimed at assessing the critical metabolic pathways altered with disease severity in hospitalized COVID-19 patients. Forty-nine hospitalized patients with COVID-19 pneumonia were enrolled in a prospective, observational, single-center study in Barcelona, Spain. Demographic, clinical, and analytical data at admission were registered. Plasma samples were collected within the first 48 h following hospitalization. Patients were stratified based on the severity of their evolution as moderate (N = 13), severe (N = 10), or critical (N = 26). A panel of 221 biomarkers was measured by targeted metabolomics in order to evaluate metabolic changes associated with subsequent disease severity. Our results show that obesity, respiratory rate, blood pressure, and oxygen saturation, as well as some analytical parameters and radiological findings, were all associated with disease severity. Additionally, ceramide metabolism, tryptophan degradation, and reductions in several metabolic reactions involving nicotinamide adenine nucleotide (NAD) at inclusion were significantly associated with respiratory severity and correlated with inflammation. In summary, assessment of the metabolomic profile of COVID-19 patients could assist in disease severity stratification and even in guiding clinical decisions.

## 1. Introduction

COVID-19 has overwhelmed health system resources worldwide. At the time of writing, it has caused more than 1,900,000 deaths [1]. A substantial proportion of patients with COVID-19 need the highest level of care in the intensive care unit (ICU) due to the rapid and unpredictable evolution towards acute respiratory distress syndrome (ARDS), the highest degree of severity. 

Early recognition of severe forms is absolutely essential for timely triaging of patients. While the clinical status, in particular, peripheral oxygen saturation levels, and concurrent comorbidities of COVID-19 patients largely determine the need for their admittance to the ICU, several laboratory parameters may facilitate the assessment of disease severity. Low lymphocyte count and serum levels of C-reactive protein (CRP), D-dimer, ferritin, cardiac troponin, and IL-6 may be used in risk stratification to predict severe and fatal COVID-19 in hospitalized patients [2]. Metabolic alterations that are intensified with disease severity might help in the early recognition of severe forms.

Viral infections are known to cause important alterations in the host metabolome, inducing molecular changes in several metabolic families including lipids and immune and energy mediators. Lipid synthesis and signaling are hijacked by viruses in order to produce lipids for their viral envelopes which allow viral entry, its release, and replication [3]. Among the immune mediators, both the kynurenine pathway and the hypothalamic-pituitary–adrenal axis have been reported to be activated in viral and bacterial infections [4,5].

Recently, some studies applied metabolomic strategies to evaluate the plasma and/or serum changes in the metabolome of COVID-19-positive patients and the same pathways described before in other viral infections seem to be altered. Most of them compared patients with healthy controls [6,7,8,9,10,11], with only a few reports evaluating the metabolic differences between mild and severe cases [7,8]. Recent publications identified metabolomic differences among healthy controls and SARS-CoV-2-positive patients. However, there is a paucity of data regarding metabolic alterations among COVID-19 patients with high disease severity.

We hypothesized the existence of a specific metabolic signature at hospital admission in hospitalized COVID-19 patients able to reveal the subsequent disease severity, helping the patients’ stratification. Consequently, this study aimed to elucidate the metabolic processes altered with disease severity in hospitalized COVID-19 patients with pneumonia. For that purpose, plasmatic levels of markers from metabolic pathways previously reported to be altered by viral infections (lipid disposition, immune and energy mediators) were measured in moderate, severe, and critical COVID-19 patients within the first 48 h after admission. Additionally, we evaluated the correlations between metabolites and clinical data available to physicians when taking care of these patients in order to explore associations between plasma metabolites and clinically relevant indices.

## 2. Results

**Figure 1:**
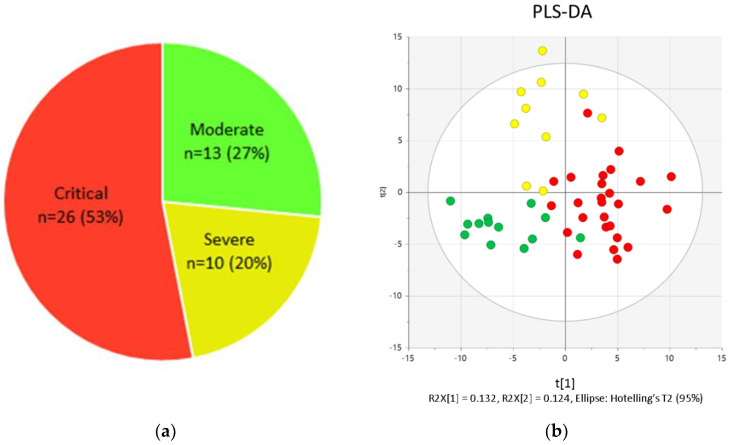
(**a**) Distribution of hospitalized COVID-19 patients stratified according to severity; (**b**) supervised partial least squares discriminant analysis (PLS-DA) showing the discrimination between COVID-19 patients regarding severity.

A total of 49 patients were included in the study. Based on their evolution, 13 patients (27%) were classified as moderate, 10 (20%) as severe, and 26 (53%) as critical (Figure 1a). Various metabolic fingerprints allowed for the separation of the three groups (Figure 1b). Among them, the most relevant markers for separation by severity were metabolites related to tryptophan metabolism, ceramides, ratios involved in NAD-consuming reactions, steroids, and lipids (Supplementary Material, Figure S1).

### 2.1. Characteristics of Patients Based on Severity

The demographic and clinical characteristics of COVID-19 patients with pneumonia included in the study and stratified by severity (moderate, severe, and critical) are shown in Table 1. Briefly, the prevalence of obesity was higher in the critical patient group than in the severe and moderate groups (*p* = 0.042 and *p* = 0.012) and their oxygen saturation at inclusion was lower (*p* = 0.001 and *p* < 0.001, respectively). Bilateral infiltrates were more prevalent in the critical and severe groups than in the moderate group (*p* < 0.001 and *p* = 0.012, respectively) and they presented more tachypnea (*p* = 0.020 and *p* = 0.005). When compared to the moderate group, the critical and severe groups more frequently received dexamethasone (*p* < 0.001 and *p* = 0.001) before inclusion and had a more severe lymphocytopenia (*p* < 0.001 and *p* = 0.005, respectively) and higher levels of D-dimer (*p* = 0.001 and *p* = 0.025) and LDH (*p* < 0.001 and *p* = 0.002) at inclusion. Patients in the critical group presented dyspnea more frequently as a symptom (*p* < 0.001), showed lower diastolic and mean blood pressure (*p* = 0.045 and *p* = 0.006), and their levels of CRP and fibrinogen were higher (*p* < 0.001 and *p* = 0.027) than in the moderate group. Patients in the critical group showed higher mortality (0% vs. 0% vs. 30.8%; *p* = 0.015) and longer hospital stay (6 (2–9) vs. 12 (8–19) vs. 34 (23–49) days; *p* ≤ 0.001) than patients in the moderate and severe groups, respectively.

Non-obese and obese patients were compared in each group to elucidate the role of obesity in the metabolomic findings. Supplementary Table S1 shows the clinical characteristics between non-obese and obese patients in each severity group, with the majority of obese patients being included in the critical group. Obese critical group patients showed a higher prevalence of dyslipidemia and longer time from first symptom until inclusion (GAP symptoms) (*p* = 0.001 and *p* = 0.014).

### 2.2. Metabolomics Results

In order to obtain alterations associated with severity, biomarkers showing a significant increase/decrease from moderate to critical were considered. Metabolites showing differences between moderate and severe groups in which this trend was not confirmed with the critical group were discarded. Among the 221 targeted biomarkers, only ceramides, tryptophan metabolism, and NAD-consuming reactions showed a clear increase/decrease in regard with the three severity groups. 

**Figure 2:**
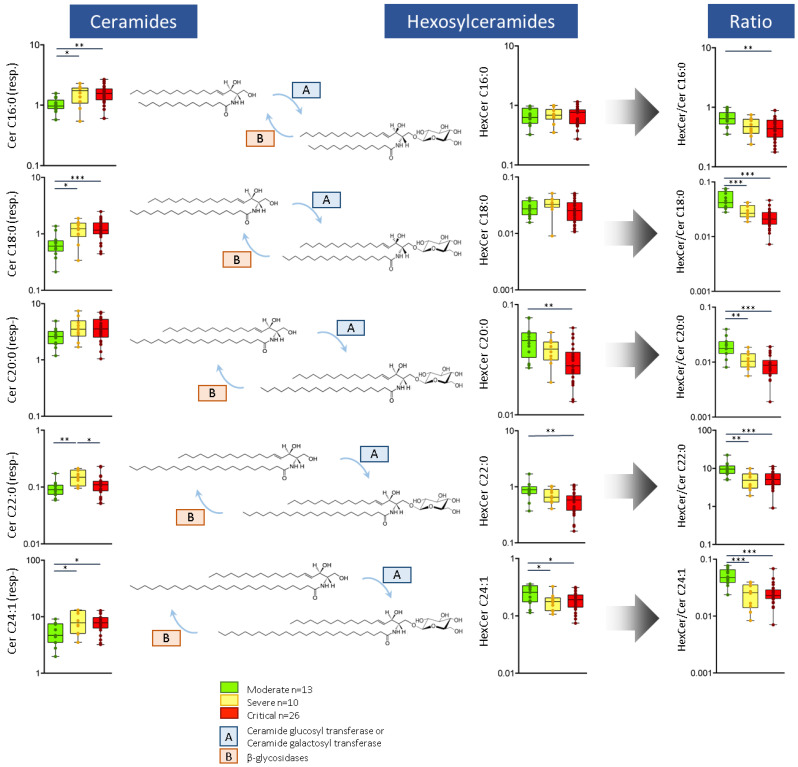
Plasma metabolic levels of long-chain ceramides (in response), hexosylceramides (in response), and hexosylceramide/ceramide ratios in hospitalized patients with COVID-19. Abbreviations: Cer: ceramides; HexCer: hexosylceramides, resp.: response. * *p* ≤ 0.05, ** *p* ≤ 0.01, *** *p* ≤ 0.001.

Metabolomic analysis revealed that levels of plasma ceramides increased with disease severity (Figure 2). This was most clearly observed in ceramides C18:0, C16:0, and C24:1. In contrast, plasma levels of very long hexosylceramides decreased with severity (HexCer C20:0, HexCer C22:0, HexCer C24:0, HexCer C24:1). Consequently, the hexosylceramide/ceramide ratio was significantly altered in all ceramide metabolites (*p* < 0.005), with lower plasma concentrations in severe and critical patients compared to the moderate illness group. Detailed mean values ± SD of all individual ceramides, hexosylceramides, and ratios are presented in Supplementary Table S2.

**Figure 3:**
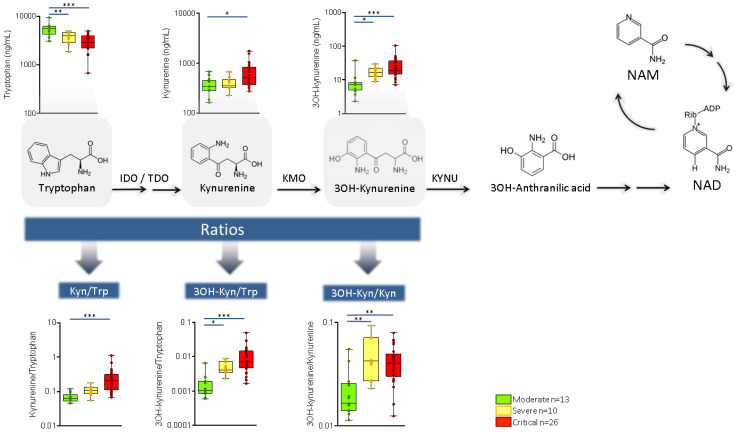
Metabolic pathway of the tryptophan metabolism via the kynurenine pathway in hospitalized patients with COVID-19 pneumonia. Abbreviations: IDO/TDO: indoleamine dioxygenase; KAT: kynurenine aminotransferase; KMO: kynurenine hydroxylase; KYNU: kynureninase; NAM: nicotinamide; NAD: nicotinamide adenine dinucleotide; NicA: nicotinic acid. * *p* ≤ 0.05, ** *p* ≤ 0.01, *** *p* ≤ 0.001.

Plasma levels of tryptophan and some of its metabolites via the kynurenine pathway also varied in accordance with COVID-19 severity (Figure 3). Specifically, tryptophan concentrations significantly decreased with disease severity (*p* ≤ 0.001) whereas several of the kynurenine pathway metabolites increased. This was observed, above all, in kynurenine (*p* = 0.012) and 3-hydroxykynurenine (*p* ≤ 0.001). Thus, kynurenine/tryptophan, 3-hydroxykynurenine/kynurenine, and 3-hydroxykynurenine/tryptophan significantly increased with COVID-19 severity (*p* ≤ 0.001). More details are shown in Supplementary Table S2.

**Figure 4:**
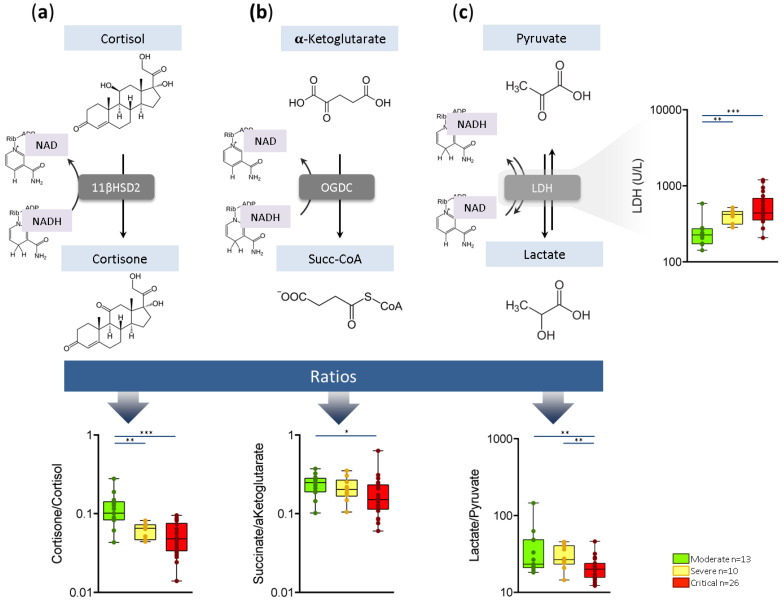
Metabolic pathways requiring NAD stratified according to the COVID-19 severity are represented by the (**a**) conversion of cortisol into cortisone via 11βHSD; (**b**) conversion of α-ketoglutarate to succinate via OGDC; and (**c**) production of LDH (estimated by the conversion of pyruvate into lactate). Abbreviations: 11βHSD: 11β-hydroxysteroid dehydrogenase; LDH: lactate dehydrogenase; NAD: nicotinamide adenine dinucleotide; OGDC: 2-oxoglutarate decarboxylase; Succ-CoA: succinyl-CoA. * *p* ≤ 0.05, ** *p* ≤ 0.01, *** *p* ≤ 0.001.

Additional alterations were observed among metabolite ratios of metabolic reactions requiring nicotinamide adenine dinucleotide (NAD+) as a cofactor. The cortisone/cortisol ratio, which illustrates the efficiency of the NAD+-dependent enzyme 11β-HSD2, decreased with COVID-19 severity (*p* ≤ 0.001) (Figure 4a). The succinate/α-ketoglutarate ratio, related to the NAD+-dependent enzyme oxoglutarate dehydrogenase complex (OGCD), also varied according to disease severity (*p* = 0.041) (Figure 4b). Lactate dehydrogenase (LDH), the enzyme that catalyzes the conversion of pyruvate into lactate using NADH as a cofactor, presented differences between severity groups. Notably, LDH content increased with disease severity (*p* = 0.004) but its efficiency, illustrated by the lactate/pyruvate ratio, decreased with COVID-19 severity (*p* = 0.003) (Figure 4c).

Similar results in these pathways were obtained when adjusted by age and gender (Table S2). Additionally, similar behavior was observed when analyzing non-obese and obese patients separately, despite the differences in the groups (Supplementary Material, Figures S2 and S3). 

**Figure 5:**
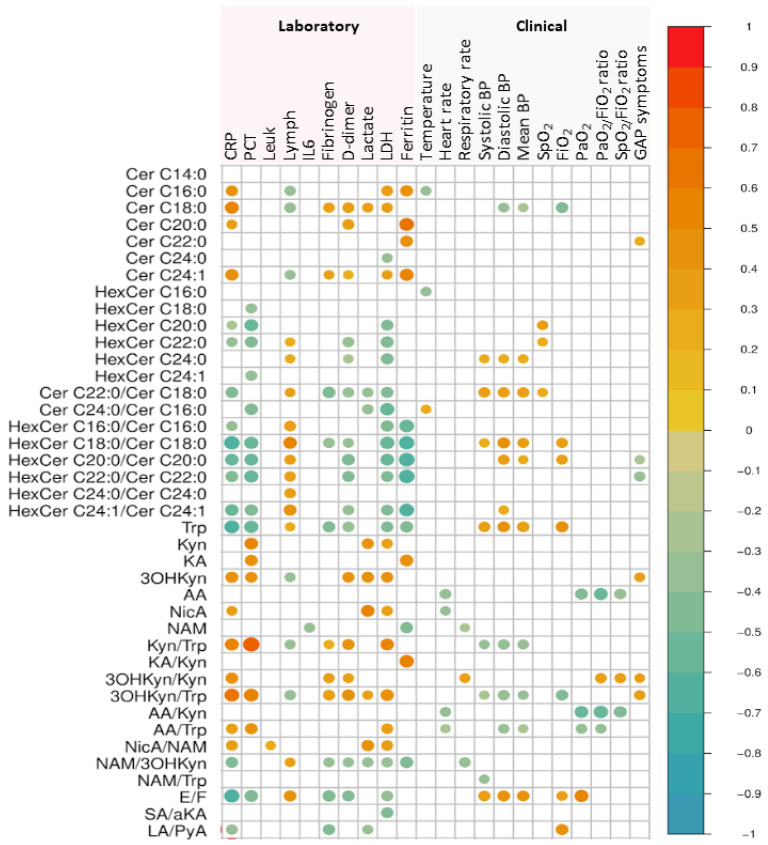
Clinical and laboratory correlates with metabolomics. Correlation plots illustrate Pearson correlations between plasma biomarkers (after log transformation) and clinical/laboratory parameters. Positive statistical significant correlations are shown in red circles and negative statistical significant correlations are shown in blue circles. The circle size represents the magnitude of the individual correlation. Abbreviations: AA: anthranillic acid; aKA: α-ketoglutarate; BP: blood pressure; Cer: ceramide; CRP: C-reactive protein; E: cortisone; F: cortisol, GAP: days from first symptom until inclusion; HexCer: hexosylceramide; 3OHKyn: 3-hydroxykynurenine; IL6: interleukin 6; KA: kynurenic acid; Kyn: kynurenine; LA: lactate; LDH: lactate dehydrogenase; Leuk: leukocytes; Lymph: lymphocytes; NAM: nicotinamide; NicA: nicotinic acid; PCT: plateletcrit; PyA: pyruvic acid; SA: succinic acid; Trp: tryptophan.

Figure 5 shows statistically significant correlations between clinically relevant parameters and metabolite pathways. In summary, plasma ceramides, hexosylceramides, and their ratios showed a statistically significant correlation with CRP, lymphocyte count, and LDH. Markers of tryptophan metabolism were also correlated with inflammatory markers and GAP symptoms. Metabolic reactions involving NAD+/NADH also correlated with laboratory and clinical parameters: both the cortisone/cortisol ratio and lactate/pyruvate ratio correlated with CRP, fibrinogen, LDH, and the fraction of inspired oxygen, whereas the succinate/α-ketoglutarate ratio correlated with LDH.

## 3. Discussion

The key findings of the present study are that alterations in ceramides, tryptophan catabolism, and NAD+ availability, together with an exacerbated pro-inflammatory response, are associated with greater severity in individuals with COVID-19 pneumonia. Additionally, several of the metabolites that were altered with increased severity correlated with clinical parameters. 

In this study, clinical variables commonly available to treating physicians in the initial care of patients with COVID-19 pneumonia were associated with disease severity, as has been suggested before [12,13]. Additionally, our results provide enhanced current evidence regarding alterations in the plasma lipidome and tryptophan catabolism related to COVID-19 severity [6,7,8,9]. We show that the levels of these biomarkers are altered at different stages of disease severity. Given the existing link between COVID-19 and obesity [14], we evaluated the potential role of obesity and found that these alterations occur irrespective of obesity, gender, or age. These metabolic alterations correlated not only with disease severity, but also with the GAP symptoms and two prognostic predictors of COVID-19 mortality: lymphopenia and CRP levels [15,16]. This finding provides further support for the role of ceramides and tryptophan both as predictive biomarkers of COVID-19 severity and as biomarkers of disease progression.

As the causative agent of COVID-19 is an enveloped virus which is surrounded by a lipid bilayer, it has been recently hypothesized that sphingolipids (including ceramides and their derivatives) are promising drug targets for the management of COVID-19 [17]. In this regard, we found COVID-19 severity to be associated with an increase in plasma ceramides and a decrease in hexosylceramides. Ceramides are bioactive lipids involved in inflammation, apoptosis, obesity, and insulin resistance, and are biomarkers of cardiovascular diseases [18]. Similarly, hexosylceramides regulate a wide variety of cell processes, including inflammation, apoptosis, and cell signaling [19]. We found that the hexosylceramide/ceramide ratio decreased with disease severity. These findings are compatible with either a decreased activity of ceramide glucosyl or galactosyl transferase (responsible for the conversion of ceramides to hexosylceramides), or a higher activity of β-glycosidases (responsible for the hydrolysis of hexosylceramides to ceramides). The alteration of this metabolic ratio is a major finding of this study and is associated with COVID-19 severity, but further research is required to elucidate its origin. To the best of our knowledge, this is the first study to report that ceramides and hexosylceramides are key metabolites that are altered in COVID-19 and that correlate with disease severity, which could be in agreement with the hypothesis described before. 

Alterations were also observed in the kynurenine pathway according to disease severity. The kynurenine pathway is involved in the *de novo* biosynthesis of the redox cofactor NAD+ which regulates the macrophage immune function in inflammation [20]. However, the over-activation of the kynurenine pathway under innate immune challenge may fail to increase NAD+ levels by limiting the conversion of quinolinate into NAD+ [20], thus producing an imbalance in NAD+ availability. We found some evidence of this imbalance, by means of the alterations shown in several metabolite ratios that require NAD+ as cofactor—for example, the transformation of cortisol into cortisone by 11βHSD2, or the transformation of pyruvate into lactate by LDH. In vitro data have shown that COVID-19 infection is sufficient to depress host cell NAD metabolism [21]. Our in vivo results are in agreement with alterations in either NAD+ production or the NAD+/NADH ratio, linked with the activity and transcription of certain viruses [22,23]. 

An increase in plasma LDH is a common feature in severe COVID-19 patients [24]. Our results confirmed the association of this increment with severity. In contrast, we observed a decrease in the lactate/pyruvate ratio (the product and precursor of the reaction catalyzed by LDH) in critical patients. The increase in LDH observed in severe and critical patients might be due to tissue injury and/or an attempt to promote the anaerobic production of energy. However, the lower lactate/pyruvate ratio values shown in critical patients indicate that this increase in LDH activity was not translated into higher levels of lactate. Then, alterations in the cofactor (either NAD+ production or NAD+/NADH ratio) are the most feasible reasons for that imbalance.

Previous metabolomics studies revealed alterations in the tryptophan catabolism and lipid disposition in COVID-19 patients [7,8,9,10,11]. Our results not only confirm the relevance of these pathways in the disease but also suggest a relationship between some of these alterations and severity. In this regard, our study shows that the increase in kynurenine previously reported in severe COVID-19 cases [7] is even stronger in other metabolites of pathways, such as 3-hydroxykynurenine, supporting the key role of NAD+ production in COVID-19 severity. Our data also support this role by the study of several metabolic ratios. Additionally, previous metabolic studies showed enrichment in some ceramide derivatives (monosialodihexosyl gangliosides) in isolated exosomes with disease severity [8]. Our study shows that this increase is also observed in plasmatic ceramides and that it is followed by a decrease in hexosylceramides.

The present study has both strengths and limitations. Some of the strengths include the exhaustive clinical and biochemical characterization of COVID-19 patients with pneumonia, as well as their classification in three different severity categories, including moderate, severe, and critical cases. Secondly, a total of 221 metabolomic biomarkers were measured in all the study individuals using a targeted metabolomics approach including six different analytical methods and using small amounts of plasma (200 µL in total). The use of these validated methods allowed for producing quantitative data and establishing metabolic ratios. Despite these strengths, we acknowledge some limitations. First, the sample size is small, and so the results need to be interpreted with caution. Second, the timing of sample collection was important to the study objectives, and so all samples were collected within the first 48 h of hospital admission but, unfortunately, the exact time of sample collection was not recorded. Third, while important metabolomics alterations have been found to correlate with severity, we cannot rule out the possibility that additional biomarkers which were not measured due to our targeted approach are additionally altered with COVID-19 severity. 

## 4. Materials and Methods

### 4.1. Study Design

This study is a prospective, observational, single-center study designed and conducted in accordance with the amended Declaration of Helsinki and approved by the Institutional Review Board of the PSMAR (Hospital del Mar, Barcelona, Spain, number 2020/9191/I). Due to the pandemic situation, patients or their next of kin gave verbal informed consent to participate for all participants. After hospital discharge, patients who survived signed a written informed consent.

### 4.2. Patients and Selection Criteria

Patients between 18 and 70 years old with a diagnosis of pneumonia and polymerase chain reaction (PCR) positive for COVID-19 admitted to the university general hospital during the first wave of the pandemic (10 March to 30 May 2020) were included. Patients with a “do not resuscitate order” were excluded. 

### 4.3. Variables Analyzed

The following information was collected from the electronic medical records: demographic details (age, sex), body mass index (BMI), illness severity (APACHE II), comorbidities (e.g., obesity (if BMI was higher than 30 Kg/m^2^)) and chronic medications, symptoms on admission, days from first symptom until inclusion (GAP symptoms), treatments received in the hospital prior to inclusion, and vital signs, laboratory test results, and radiological findings at inclusion. Laboratory parameters measured at inclusion were C-reactive protein (CRP, mg/dL), procalcitonin (ng/mL), interleukin 6 (IL-6; pg/mL), lymphocytes (103 cells/µL), leukocytes (103 cells/µL), fibrinogen (mg/dL), lactate mg/dL, lactate dehydrogenase (LDH, U/L) ferritin (µg/dL), and D-dimer (ng/mL). Length of hospital stay (days) and hospital mortality were also recorded.

### 4.4. Study Groups

Patients’ severity was retrospectively evaluated at hospital discharge. Patients were classified into different groups according to their severity based on previous literature [25]: (1) moderate, when they showed fever and respiratory symptoms; (2) severe, when any of the following criteria were present: respiratory distress (>30 breaths/min) or oxygen saturation <93% at rest or arterial pressure of oxygen (PaO_2_)/fraction of inspired oxygen (FIO_2_) (PaO_2_/FIO_2_ ratio) <300 mmHg; and (3) critical when cases presented with hypoxemic respiratory failure requiring mechanical ventilation, or shock or other organ failure requiring ICU care [25].

Due to the low severity of the disease in the moderate group, some clinical data in this group could not be collected, for example, FIO_2_ (because most patients were under nasal prongs) or PaO_2_ (because no arterial blood gases were needed). 

### 4.5. Sample Collection and Metabolomics

Plasma samples from all COVID-19 patients were collected within 48 h of hospital admission. After plasma inactivation, six protocols were followed for each family of compounds (carboxylic acids, polar neurotransmitters, lipids, kynurenine pathway, and steroids) using previously reported methods [26,27,28,29,30] and specific details are provided in the Supplementary Material. A panel of 221 targeted biomarkers (Supplementary Table S3) was measured by liquid chromatography coupled to tandem mass spectrometry (LC–MS/MS) consisting of an Acquity UPLC instrument (Waters Associates, Milford, MA, USA) coupled to a triple quadrupole (TQS Micro, Waters) mass spectrometer. Targeted analytes were determined by selected reaction monitoring. MassLynx software V4.1 (Waters Associates) was used for peak integration and data management. The standards and reagents used and their corresponding suppliers are listed in Supplementary Table S4. 

### 4.6. Statistical Analysis

Continuous variables are expressed as the mean and standard deviation (SD) or median and interquartile range (IQR) depending on their parametric or non-parametric distribution, unless otherwise specified. Categorical variables are reported as absolute frequencies and percentages. Chi-squared or Fisher’s exact tests were used to analyze differences in all groups in terms of categorical variables. For continuous variables exhibiting a normal distribution, differences between severity groups were analyzed using one-way ANOVA and subsequently Bonferroni’s post hoc test. Otherwise, differences between severity groups were analyzed using a Kruskal–Wallis test and subsequently Dunn’s multiple comparison test. Univariable analysis and multivariable linear regression models adjusted by age and gender were used to study the effect of disease severity on the levels of metabolites. All data were analyzed using SPSS (version 22.0), R (version 4.0.2), RStudio (version 1.2.1335), and Metaboanalyst (version 3.0) software. Comparisons were considered to be statistically significant when the level of significance was *p* < 0.05. Additionally, an exhaustive automated exploratory data analysis process (AutoDiscovery) was performed. Further details are provided in the Supplementary Material.

## 5. Conclusions

The metabolomic profile of COVID-19 patients at admission may be useful for determining their evolution. Pathways related to ceramides, tryptophan, and NAD production, together with an exacerbated pro-inflammatory response, seem to be the mechanisms most closely linked to severity. An assessment of these pathways may be useful for supporting clinical decisions in the management of severe and critically ill patients in the ICU.

## Figures and Tables

**Table 1:** Characteristics of hospitalized COVID-19 patients included in the study by severity.

	All Patients (N = 49)	Moderate (N = 13)	Severe (N = 10)	Critical (N = 26)
**Demographics**				
Age, years	55 (14)	51 (18)	50 (14)	59 (10)
Gender, male n (%)	24 (49)	7 (53.8)	5 (50)	12 (46.2)
Body mass index, Kg/m^2^	30 (7)	25 (6)	27 (4)	32 (7)
APACHE II score ^a^	13 (6)	6 (3)	11 (5) ^b^	16 (6) ^b,c^
**Chronic comorbidities n (%)**	28 (57.1)	4 (30.8)	6 (60)	18 (69.2)
Chronic lung disease	5 (10.2)	1 (7.7)	3 (30)	1 (3.8)
Asthma	1 (2)	0 (0)	1 (10)	0 (0)
COPD	3 (6.1)	1 (7.7)	1 (10)	1 (3.8)
Cardiovascular disease Coronary artery disease	21 (42.9)	3 (23.1)	3 (30)	15 (57.7)
	1 (2)	1 (7.7)	0 (0)	0 (0)
Hypertension	21 (42.9)	3 (23.1)	3 (30)	15 (57.7)
Atrial fibrillation	1 (2)	0 (0)	0 (0)	1 (3.8)
Other medical conditions				
Immunosuppression	3 (6.1)	0 (0)	1 (10)	2 (7.7)
Alcoholism	7 (14.3)	3 (23.1)	1 (10)	3 (11.5)
Current or former smoker	9 (18.4)	2 (15.4)	1 (10)	6 (23.1)
Dyslipidemia	11 (22.4)	1 (7.7)	2 (20)	8 (30.8)
Diabetes mellitus	10 (20.4)	1 (7.7)	2 (20)	7 (26.9)
Liver disease	1 (2)	1 (7.7)	0 (0)	0 (0)
Chronic renal failure	3 (6.1)	0 (0)	0 (0)	3 (11.5)
Hematological malignancies	1 (2)	1 (7.7)	0 (0)	0 (0)
Solid tumor	3 (6.1)	1 (7.7)	1 (10)	1 (3.8)
Hypothyroidism	3 (6.1)	1 (7.7)	0 (0)	2 (7.7)
Obesity ^a^	19 (38.8)	2 (15.4)	2 (20)	15 (57.7) ^b,c^
HIV	1 (2)	1 (7.7)	0 (0)	0 (0)
**Chronic medications n (%)**				
Inhaled corticosteroids	1 (2)	0 (0)	0 (0)	1 (4)
Biological drugs	1 (2)	0 (0)	0 (0)	1 (4)
ACE inhibitors	9 (18.4)	1 (7.7)	2 (20)	6 (23.1)
Angiotensin II blockers	4 (8.2)	1 (7.7)	0 (0)	3 (11.5)
Statins	4 (8.2)	1 (7.7)	0 (0)	3 (11.5)
Oral corticosteroids	3 (6.1)	0 (0)	1 (10)	2 (7.7)
**Symptoms n (%)**				
Cough	36 (73.5)	8 (61.5)	9 (90)	19 (73.1)
Fever	40 (81.6)	8 (61.5)	9 (90)	23 (88.5)
Sore throat	1 (2)	1 (7.7)	0 (0)	0 (0)
Headache	9 (18.4)	4 (30.8)	3 (30)	2 (7.7)
Rhinorrhea	1 (2)	0 (0)	1 (10)	0 (0)
Myalgia	10 (20.4)	4 (30.8)	4 (40)	2 (7.7)
Sputum	7 (14.3)	0 (0)	3 (30)	4 (15.4)
Diarrhea	10 (20.4)	3 (23.1)	2 (20)	5 (19.2)
Chest pain	6 (12.2)	2 (15.4)	1 (10)	3 (11.5)
Nausea/vomiting	3 (6.1)	1 (7.7)	1 (10)	1 (3.8)
Dyspnea ^a^	30 (61.2)	3 (23.1)	6 (60)	21 (80.8) ^b^
Altered mental status	2 (4.1)	0 (0)	0 (0)	2 (7.7)
GAP symptoms, days	6 (4–9)	5 (4–8)	7 (4–11)	7 (5–9)
**Vital signs at inclusion**				
Temperature, °C	36.2 (1.2)	36.7 (1.2)	36.6 (1.1)	36.7 (1.1)
Heart rate, bpm	80(14)	82 (18)	81 (13)	78 (13)
Respiratory rate, rpm ^a^	24 (21–30)	22 (17–24)	28 (23–36) ^b^	25 (22–33) ^b^
Systolic blood pressure, mmHg	115 (21)	125 (17)	113 (19)	110 (23)
Diastolic blood pressure, mmHg ^a^	69 (16)	78 (13)	70 (14)	63 (15) ^b^
Mean blood pressure, mmHg ^a^	84 (16)	94 (13)	85 (14)	79 (16) ^b^
Oxygen saturation, % ^a^	97 (94–98)	98 (97–98)	98 (96–99)	94 (93–97) ^b,c^
Fraction of inspired oxygen, %	100 (60–100)	---	100 (46–100)	90 (60–100)
Arterial oxygen pressure, mmHg	86.8 (71.3–105.2)	---	102 (79–134)	83 (67–95)
PaO_2_/FIO_2_ ratio	100 (68–145)	---	106 (80–287)	107 (74–153)
SaO_2_/FIO_2_ ratio	99 (96–157)	---	99 (98–276)	106 (94–157)
**Analytical variables at inclusion**				
C-reactive protein, mg/mL ^a^	16.5 (13.2)	4.0 (4.0)	14.1 (10.1) ^b^	23.1 (12.8) ^b,c^
Procalcitonin, ng/mL	0.29 (0.11–0.78)	0.12 (0.04–0.13)	0.24 (0.11–0.55)	0.38 (0.24–1.13)
Leukocytes, ×10^3^/µL	7.39 (6.14–10.54)	6.33 (5.85–8.96)	6.99 (5.91–8.88)	8.89 (6.71–12.90)
Lymphocytes, ×10^3^/µL ^a^	0.94 (0.70–1.27)	1.28 (0.99–2.45)	0.84 (0.80–1.00) ^b^	0.78 (0.48–1.10) ^b^
IL-6, pg/mL	96.2 (22.8–180.15)	---	45.6 (4.7–160.7)	132.1 (22.8–216.2)
Fibrinogen, mg/dL ^a^	500 (500–500)	497 (369–500)	500 (466–500)	500 (500–500) ^b^
D-dimer, ng/mL ^a^	1200 (725–2517)	705 (395–892)	1425 (767–3532) ^b^	1660 (1070–2682) ^b^
Lactate, mg/dL	1.39 (1.01–1.85)	1.13 (0.88–1.33)	1.34 (0.94–1.87)	1.54 (1.20–2.00)
LDH, U/L ^a^	388 (275–506)	225 (173–271)	417 (313–460) ^b^	437 (353–683) ^b^
Ferritin, µg/L	841 (661–2568)	---	1009 (664–2040)	841 (556–2999)
**Radiological findings at inclusion n (%)**				
Bilateral infiltrates ^a^	43 (87.8)	7 (53.8)	10 (100) ^b^	26 (100) ^b^
Ground glass	29 (59.2)	9 (69.2)	17 (65.4)	13 (50)
Consolidation	34 (69.4)	9 (69.2)	8 (80)	
**Treatment received in hospital before inclusion n (%)**				
Hydroxychloroquine	19 (38.8)	4 (30.8)	5 (50)	10 (38.5)
Systemic corticosteroids ^a^	11 (22.4)	0 (0)	5 (50)	6 (23.1) ^b^
Tocilizumab	4 (8.2)	0 (0)	2 (20)	2 (7.7)

^a^*p* < 0.05 between moderate, severe, and critical; ^b^ *p* < 0.05 compared to moderate; ^c^ *p* < 0.05 compared to severe. Data expressed as frequencies and percentages (n (%)) or mean (SD) or median (IQR). In bold the header that comprises the variables that appear below. Abbreviations: APACHE II: acute physiology and chronic health evaluation II; COPD: chronic obstructive pulmonary disease; HIV: human immunodeficiency virus; ACE: angiotensin-converting enzyme; Symptoms gap: days from first symptom until inclusion; IL-6: interleukin 6; LDH: lactate dehydrogenase; PaO_2_/FIO_2_ ratio: arterial oxygen pressure/fraction of inspired oxygen; SaO_2_/FIO_2_ ratio: oxygen saturation/fraction of inspired oxygen.

## Data Availability

The data that support the findings of this study are available from the corresponding author upon reasonable request.

## Supplementary Materials

The following are available online at https://www.mdpi.com/article/10.3390/ijms22094794/s1.

## References

[1] Dong E., Du H., Gardner L. (2020). An interactive web-based dashboard to track COVID-19 in real time. Lancet Infect. Dis..

[2] Velavan T.P., Meyer C.G. (2021). COVID-19: A PCR-defined pandemic. Int. J. Infect. Dis..

[3] Abu-Farha M., Thanaraj T.A., Qaddoumi M.G., Hashem A., Abubaker J., Al-Mulla F. (2020). The Role of Lipid Metabolism in COVID-19 Virus Infection and as a Drug Target. Int. J. Mol. Sci..

[4] Gaelings L., Söderholm S., Bugai A., Fu Y.-K., Nandania J., Schepens B., Lorey M.B., Tynell J., Ginste L.V., Le Goffic R. (2017). Regulation of kynurenine biosynthesis during influenza virus infection. FEBS J..

[5] Silverman M.N., Pearce B.D., Biron C.A., Miller A.H. (2005). Immune Modulation of the Hypothalamic-Pituitary-Adrenal (HPA) Axis during Viral Infection. Viral Immunol..

[6] Bruzzone C., Bizkarguenaga M., Gil-Redondo R., Diercks T., Arana E., de Vicuña A.G., Seco M., Bosch A., Palazón A., Juan I.S. (2020). SARS-CoV-2 Infection Dysregulates the Metabolomic and Lipidomic Profiles of Serum. iScience.

[7] Shen B., Yi X., Sun Y., Bi X., Du J., Zhang C., Quan S., Zhang F., Sun R., Qian L. (2020). Proteomic and Metabolomic Characterization of COVID-19 Patient Sera. Cell.

[8] Song J.-W., Lam S.M., Fan X., Cao W.-J., Wang S.-Y., Tian H., Chua G.H., Zhang C., Meng F.-P., Xu Z. (2020). Omics-Driven Systems Interrogation of Metabolic Dysregulation in COVID-19 Pathogenesis. Cell Metab..

[9] Thomas T., Stefanoni D., Reisz J.A., Nemkov T., Bertolone L., Francis R.O., Hudson K.E., Zimring J.C., Hansen K.C., Hod E.A. (2020). COVID-19 infection alters kynurenine and fatty acid metabolism, correlating with IL-6 levels and renal status. JCI Insight.

[10] Blasco H., Bessy C., Plantier L., Lefevre A., Piver E., Bernard L., Marlet J., Stefic K., Bretagne I.B.-D., Cannet P. (2020). The specific metabolome profiling of patients infected by SARS-COV-2 supports the key role of tryptophan-nicotinamide pathway and cytosine metabolism. Sci. Rep..

[11] Barberis E., Timo S., Amede E., Vanella V.V., Puricelli C., Cappellano G., Raineri D., Cittone M.G., Rizzi E., Pedrinelli A.R. (2020). Large-Scale Plasma Analysis Revealed New Mechanisms and Molecules Associated with the Host Response to SARS-CoV-2. Int. J. Mol. Sci..

[12] Grasselli G., Zangrillo A., Zanella A., Antonelli M., Cabrini L., Castelli A., Cereda D., Coluccello A., Foti G., Fumagalli R. (2020). Baseline Characteristics and Outcomes of 1591 Patients Infected with SARS-CoV-2 Admitted to ICUs of the Lombardy Region, Italy. JAMA.

[13] Li L., Huang T., Wang Y., Wang Z., Liang Y., Huang T., Zhang H., Sun W., Wang Y. (2020). COVID-19 patients’ clinical characteristics, discharge rate, and fatality rate of meta-analysis. J. Med. Virol..

[14] Ritter A., Kreis N.-N., Louwen F., Yuan J. (2020). Obesity and COVID-19: Molecular Mechanisms Linking both Pandemics. Int. J. Mol. Sci..

[15] Huang I., Pranata R. (2020). Lymphopenia in severe coronavirus disease-2019 (COVID-19): Systematic review and meta-analysis. J. Intensiv. Care.

[16] Yan L., Zhang H.-T., Goncalves J., Xiao Y., Wang M., Guo Y., Sun C., Tang X., Jing L., Zhang M. (2020). An interpretable mortality prediction model for COVID-19 patients. Nat. Mach. Intell..

[17] Prakash H., Upadhyay D., Bandapalli O.R., Jain A., Kleuser B. (2021). Host sphingolipids: Perspective immune adjuvant for controlling SARS-CoV-2 infection for managing COVID-19 disease. Prostaglandins Other Lipid Mediat..

[18] Havulinna A.S., Sysi-Aho M., Hilvo M., Kauhanen D., Hurme R., Ekroos K., Salomaa V., Laaksonen R. (2016). Circulating Ceramides Predict Cardiovascular Outcomes in the Population-Based FINRISK 2002 Cohort. Arterioscler. Thromb. Vasc. Biol..

[19] Lopes-Virella M.F., Baker N.L., Hunt K.J., Hammad S.M., Arthur J., Virella G., Klein R.L. (2019). Glycosylated sphingolipids and progression to kidney dysfunction in type 1 diabetes. J. Clin. Lipidol..

[20] Minhas P.S., Liu L., Moon P.K., Joshi A.U., Dove C., Mhatre S., Contrepois K., Wang Q., Lee B.A., Coronado M. (2019). Macrophage de novo NAD+ synthesis specifies immune function in aging and inflammation. Nat. Immunol..

[21] Heer C.D., Sanderson D.J., Voth L.S., Alhammad Y.M., Schmidt M.S., Trammell S.A., Perlman S., Cohen M.S., Fehr A.R., Brenner C. (2020). Coronavirus infection and PARP expression dysregulate the NAD metabolome: An actionable component of innate immunity. J. Biol. Chem..

[22] Dickherber M.L., Garnett-Benson C. (2019). NAD-linked mechanisms of gene de-repression and a novel role for CtBP in persistent adenovirus infection of lymphocytes. Virol. J..

[23] Yu J.-W., Sun L.-J., Liu W., Zhao Y.-H., Kang P., Yan B.-Z. (2013). Hepatitis C virus core protein induces hepatic metabolism disorders through down-regulation of the SIRT1–AMPK signaling pathway. Int. J. Infect. Dis..

[24] Zhou F., Yu T., Du R., Fan G., Liu Y., Liu Z., Xiang J., Wang Y., Song B., Gu X. (2020). Clinical course and risk factors for mortality of adult inpatients with COVID-19 in Wuhan, China: A retrospective cohort study. Lancet.

[25] Wei P.F. (2020). Released by National Health Commission & National Administration of Traditional Chinese Medicine on March 3, 2020) Diagnosis and Treatment Protocol for Novel Coronavirus Pneumonia (Trial Version 7). Chin. Med. J..

[26] Gomez-Gomez A., Soldevila A., Pizarro N., Andreu-Fernandez V., Pozo O.J. (2019). Improving liquid chromatography-tandem mass spectrometry determination of polycarboxylic acids in human urine by chemical derivatization. Comparison of o-benzyl hydroxylamine and 2-picolyl amine. J. Pharm. Biomed. Anal..

[27] Marcos J., Renau N., Valverde O., Aznar-Laín G., Gracia-Rubio I., Gonzalez-Sepulveda M., Pérez-Jurado L.A., Ventura R., Segura J., Pozo O.J. (2016). Targeting tryptophan and tyrosine metabolism by liquid chromatography tandem mass spectrometry. J. Chromatogr. A.

[28] Olesti E., Rodríguez-Morató J., Gomez-Gomez A., Ramaekers J.G., De La Torre R., Pozo O.J. (2019). Quantification of endogenous neurotransmitters and related compounds by liquid chromatography coupled to tandem mass spectrometry. Talanta.

[29] Gomez-Gomez A., Miranda J., Feixas G., Betegon A.A., Crispi F., Gratacós E., Pozo O.J. (2020). Determination of the steroid profile in alternative matrices by liquid chromatography tandem mass spectrometry. J. Steroid Biochem. Mol. Biol..

[30] Pozo O.J., Marcos J., Khymenets O., Pranata A., Fitzgerald C.C., McLeod M.D., Shackleton C. (2018). Sulfation pathways: Alternate steroid sulfation pathways targeted by LC–MS/MS analysis of disulfates: Application to prenatal diagnosis of steroid synthesis disorders. J. Mol. Endocrinol..

