# Supplementary material for: Metabolic Signatures Associated with Severity in Hospitalized COVID-19 Patients
Source: Int J Mol Sci. 2021 Apr 30;22(9):4794. doi: 10.3390/ijms22094794 (PMC8124482; doi:10.3390/ijms22094794)
Supplement: Supplementary file 1 [file ijms-22-04794-s001.zip › ijms-1156574-supplementary.pdf]

Metabolic signatures associated with severity in hospitalized COVID–19 patients

Online Supplementary Material

Table of contents

1. Supplementary materials and methods 2
1.1. Standards and reagents 2
1.2. Sample collection 2
1.3. Sample inactivation 2
1.4. Sample preparation for targeted metabolomics 2
1.5. Instrumentation 3
1.6. Statistical analysis 3
2. Supplementary Figures 5
2.1. Figure S1. Main markers involved in the separation between (A) critical and moderate/severe patients (t[1] in Figure 1) and (B) severe and moderate/critical patients (t[2] in Figure 1) 5
2.2. Figure S2. Plasma levels of ceramides, hexosylceramides and hexosylceramide and ceramide ratios, in non-obese and obese patients in analysed groups. 6
2.3. Figure S3. Plasma levels of metabolites included in tryptophan metabolism via the kynurenine pathway, in non-obese and obese patients in analysed groups. 7
3. Supplementary Tables 7
3.1. Table S1. Characteristics of hospitalized COVID-19 patients included in the study by severity and obesity. 8
3.2. Table S2. Sphingolipids and tryptophan metabolites in moderate, severe, and critical hospitalized COVID-19 patients. 0
3.3. Table S3. Detailed list of 221 biomarkers studied in COVID-19 patients grouped according to their corresponding pathway. 0
3.4. Table S4. Standards and reagents, and their corresponding suppliers 0
4. References 0

1. Supplementary materials and methods

1.1. Standards and reagents

The standards and reagents used in the present study were supplied by Sigma Aldrich (Saint Louis, MO, USA), Alsachim (Illkirch-Graffenstaden, France), Toronto Research Chemicals (Toronto, Canada), Cambridge Isotope Laboratories (Tewksbury, MA, USA), Merck (Darmstadt, Germany), NMI (Sydney, Australia) and Millipore Ibérica (Barcelona, Spain) as detailed in Table S4.

1.2. Sample collection

Peripheral blood samples were collected in EDTA tubes and plasma was separated by centrifugation at 3,000 rpm for 10 minutes and stored at -80ºC until further analysis.

1.3. Sample inactivation

Plasma (200 µL) was inactivated via the addition of 800 µL of ice-cold 80% methanol and following this, the protein precipitation protocol was applied to all samples as described in Supplementary material (Sample preparation for targeted metabolomics).

1.4. Sample preparation for targeted metabolomics

Plasma samples previously inactivated in ice-cold methanol were centrifuged (4500 rpm, 10 min at 4 ºC) to accomplish with protein precipitation. Each supernatant was aliquoted in 6 fractions and were stored at -80ºC until analysis.

The determination of 221 plasma biomarkers (including 125 metabolites and 96 ratios between metabolites with potential information about enzyme activity) was achieved using six different LC-MS/MS methods. Differences in the chemical structure of the analytes made necessary the application of different analytical approaches for the proper quantification of the analytes. Six different sample preparation protocols were followed for each one of the families of compounds: carboxylic acids, polar neurotransmitters and related compounds, lipids, kynurenine pathway, phase I, and phase II steroids.

1.4.1. Carboxylic acids

The analysis of carboxylic acids was performed by adapting a previously reported method.[1] Initially, 25 µL aliquot of plasma diluted x5 were mixed with 30 µL of the corresponding ISTD (Table S4). Then, carboxylic acids were derivatized by adding 100 µL of the mixture of o-benzyl hydroxylamine (1M) and N-(3-Dimethylaminopropyl)-N'-ethylcarbodiimide hydrochloride (1M) into the samples and left at room temperature during 1 hour. Subsequently, a liquid-liquid extraction with water and ethyl acetate was performed and the organic extract was evaporated until dry (N2 stream, 40 ºC, <15 psi). The extracts were reconstituted in 300 µL of water: methanol (1:1, v:v) and 3 µL were injected into the LC-MS/MS system.

1.4.2. Polar neurotransmitters and related compounds

Fourteen neurotransmitters and related compounds were quantified in the plasma samples by using two alternative treatments (dilution x5 and dilution x100), both adapted from previously reported method.[2] For the first treatment, 25 µL aliquot of plasma sample diluted x5 was mixed with 50 µL of the corresponding ISTD (Table S4) and 115 µL of acetonitrile. For the second, plasma sample aliquot was diluted with water until factor 100, and 25 µL of this dilution was mixed with 50 µL of the corresponding ISTD (Table S4) and 115 µL of acetonitrile. Both extracts were injected into the LC-MS/MS system for each matrix.

1.4.3. Lipids

A panel of five lipid families [diacylglycerols; (DAG), ceramides (Cer), hexosylceramides (HexCer), lysophosphatidylcholines (LPC), and sphingomyelin (SM)] in plasma samples were determined as follows. Fifty µL of plasma diluted x5 were spiked with 100 µL of the corresponding ISTD (Table S4). Samples were vortexed, centrifuged (5 min, 10,000 rpm), and injected into the LC-MS/MS system. Further details have been published elsewhere.[3]

1.4.4. Kynurenine pathway

Biomarkers belonging to the tryptophan metabolism were quantified following a previously reported method.[4] Briefly, 50 µL of the working standard solution (ISTD, Table S4) were added to 400 µL of plasma diluted x5 and then evaporated until dry (N_2 stream, 29 ºC, <15 psi) and reconstituted with 100 µL of water. Finally, 10 µL were injected into the LC-MS/MS instrument.

1.4.5. Phase I and phase II steroids

Steroids were determined by the adaptation of previously reported methods.[5,6] An aliquot of 350 µL of plasma diluted x5 was mixed with 10 µL of corresponding ISTD (Table S4) and 1 mL of methanol. Afterwards, samples were evaporated under stream of nitrogen at 40 ºC and reconstituted in 100 µL of water: acetonitrile (9:1, v/v). Ten µL were injected into the LC-MS/MS system.

1.5. Instrumentation

The chromatographic separation and detection of the analytes was performed using an Acquity UPLC system (Waters Associates, Milford, MA, USA) coupled to a triple quadrupole (Xevo TQs) mass spectrometer (Waters Associates) provided with an orthogonal Z-spray-electrospray interface (ESI).

The liquid chromatography (LC) separation for tryptophan metabolites, carboxylic acids, lipids and steroids (phase I) was performed using an Acquity BEH C18 column (100 mm x 2.1 mm i.d., 1.7 µm) (Waters Associates) with a flow rate of 0.3 mL/min at 55 ºC. For neurotransmitters, the LC separation was achieved by an Acquity UPLC BEH Amide 1.7 µm (100 mm x 2.1 mm i.d., 1.7 µm) (Waters Associates) at a flow rate of 0.6 mL/min at 55 ºC, and for the steroids (phase II) an Acquity UPLC CSH C18 column (2.1 × 100 mm i.d., 1.7 µm) (Waters Associates) at a flow rate of 0.4 mL/min at 30ºC was used. The mass spectrometric detection was achieved by using the positive ionization mode for tryptophan metabolites, carboxylic acids, lipids, steroids (phase I) and neurotransmitters and using the negative ionization mode for steroids (phase II).

The mobile phases selected for the determination of tryptophan metabolites, carboxylic acids, lipids and steroids (phase I) were water-ammonium formate (1 mM)-formic acid (0.01%) as mobile phase A and methanol-ammonium formate (1 mM)-formic acid (0.01%) as mobile phase B. Due to the chemical differences of each group of analytes, various chromatographic gradients were applied. The gradient program for the determination of tryptophan metabolites increased the percentage of mobile phase B linearly, as follows: 0 min, 1%; 0.5 min, 1%; 7 min, 40%; 8.5 min, 90%; 9 min, 90%; 9.5 min, 1%; 12 min, 1%. For carboxylic acid determination, the gradient program changed the percentage of mobile phase B linearly, as follows: 0 min, 30%; 1 min,30%; 6 min, 55%; 6.8 min, 80%; 8.3 min, 99%; 9 min, 99%; 9.01 min, 30%; 10 min, 30%. For lipid determination, an isocratic gradient at 100% of mobile phase B was used for 5 minutes and for the determination of steroids (phase I) a gradient program was used with a percentage of mobile phase B, changing linearly as follows: 0 min,15%; 0.5 min, 15%; 3 min, 40%; 16 min, 70%; 17 min, 90%; 18 min, 90%; 18.5 min, 15%; 20 min, 15%.

The mobile phases selected for the determination of neurotransmitters and steroids (phase II) were water-ammonium formate (25 mM)-formic acid (0.01%) as mobile phase A and acetonitrile:water (9:1)-ammonium formate (25 mM)-formic acid (0.01%) as mobile phase B. For neurotransmitters, the gradient program used changed the percentage of mobile phase A linearly, as follows: 0 min, 10%; 0.5 min, 10%; 2 min, 40%; 2.5 min, 40%; 2.6 min, 10%; 3.5 min, 10%. For steroids (phase II) separation, 25 mM ammonium formate in both water and acetonitrile:water (9:1) were used as aqueous and organic mobile phases, respectively, and the gradient program was as followed: 0 min, 10%; 0.5 min, 10%; 13 min, 43%; 13.5 min, 100%; 14 min, 100%; 14.5 min, 10%; 16 min, 10%.

1.6. Statistical analysis

An exhaustive automated exploratory data analysis process (AutoDiscovery) was performed. The degree of association between each pair of variables of interest in the study based on the exploratory goals previously defined was automatically assessed. To do this, the system selected the proper numerical method based on the data type and distribution of the variables assessed. The numerical tests applied in each case were Spearman's Rank Correlation, Variance Analysis (ANOVA one-way, Mann-Whitney U, and Kruskal-

Wallis; normality was tested in these cases with D'Agostino/Pearson methods), and Cramer's V Contingency Index. This process was performed in each of the possible subgroups of patients generated by means of the qualitative factors previously configured. Subgroups or associations with a sample size below 5, a sample size below 1% of the total sample size or a statistical significance (*p*-value) equal or higher than 0.05 were automatically rejected. Given the nature of this multiple testing method, a high significance threshold was calculated based on the Benjamini-Hochberg method (False-Discovery Rate) to classify the rest of associations. Metabolites with signal below the limit of detection of the different analytical methods used were assigned to ½ of this limit. Univariable analysis and multivariable linear regression model adjusted by age and gender were used to study the effect of disease severity on the levels of metabolites. Partial-Least Square-Discriminant Analysis (PLS-DA) and orthogonal-PLS-DA (oPLS-DA) were also used to evaluate potential metabolic differences associated with severity. Linear correlations were calculated using Pearson's test after checking for normality and log-transforming, when necessary.

2. Supplementary Figures

2.1. Figure S1. Main markers involved in the separation between critical and moderate patients (t[1] in Figure 1). Abbreviations: 3OHKyn/Trp: 3-Hydroxykynurenine / Tryptophan; HexCer/Cer18:0: Hexosylceramide C18:0 / Ceramide C18:0; Kyn/Trp: Kynurenine/Tryptophan; 3OHKyn: 3-Hydroxykynurenine; E/F: Cortisone / Cortisol; HexCer/Cer20:0: Hexosylceramide C20:0 / ceramide C20:0; Trp: Tryptophan; Isovaleric/bOH: Isovalerate / β-hydroxybutyrate; Val/Trp: Valine / Tryptophan; HexCer/Cer241: Hexosylceramide C24:1 / ceramide C24:1; Trp/LNAA: Tryptophan / Long neutral amino acids; AcAcO: Acetoacetate; AllE/AllF: Cortisone metabolites / Cortisol metabolites; Trp/Phe: Tryptophan/Phenylalanine; AcAcO/Leu: Acetoacetate/Leucine.

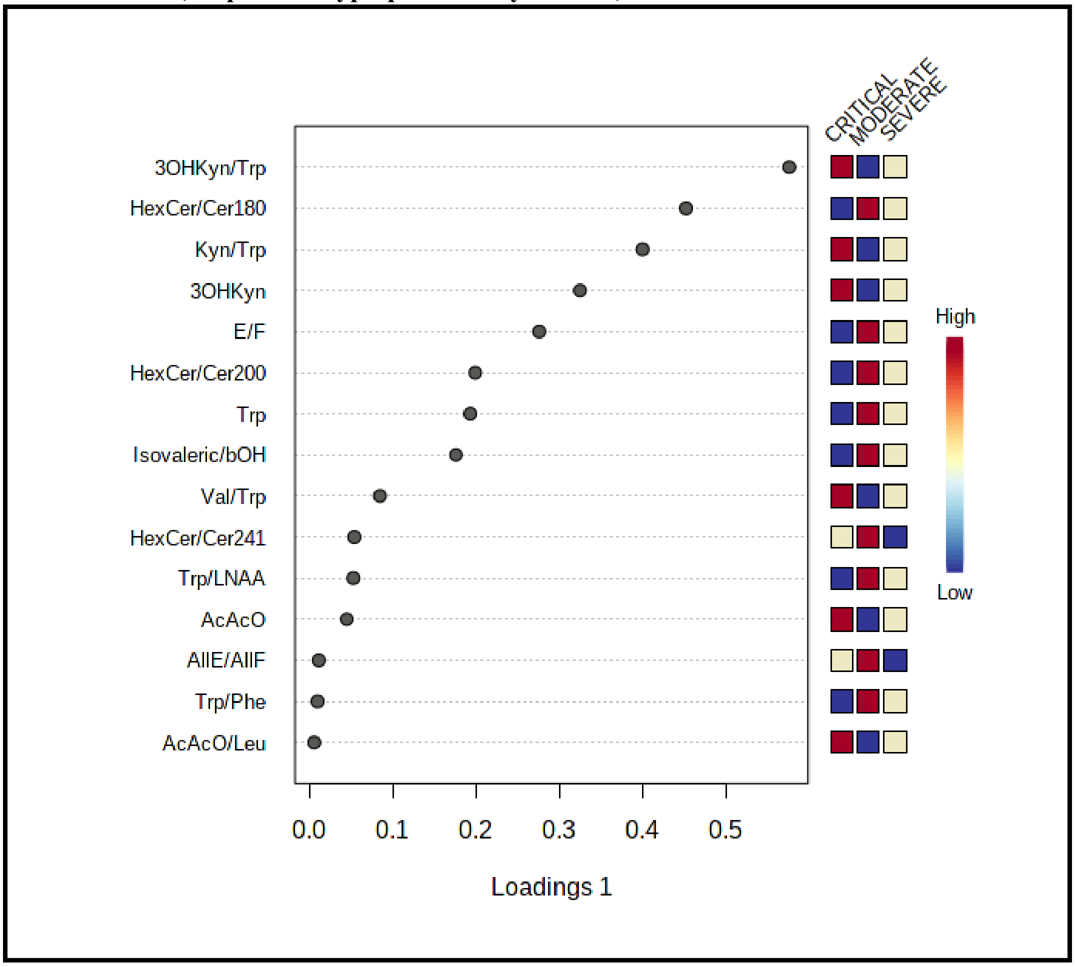

2.2. **Figure S2. Plasma levels of ceramides, hexosylceramides and hexosylceramide and ceramide ratios, in non-obese and obese patients in analysed groups.**

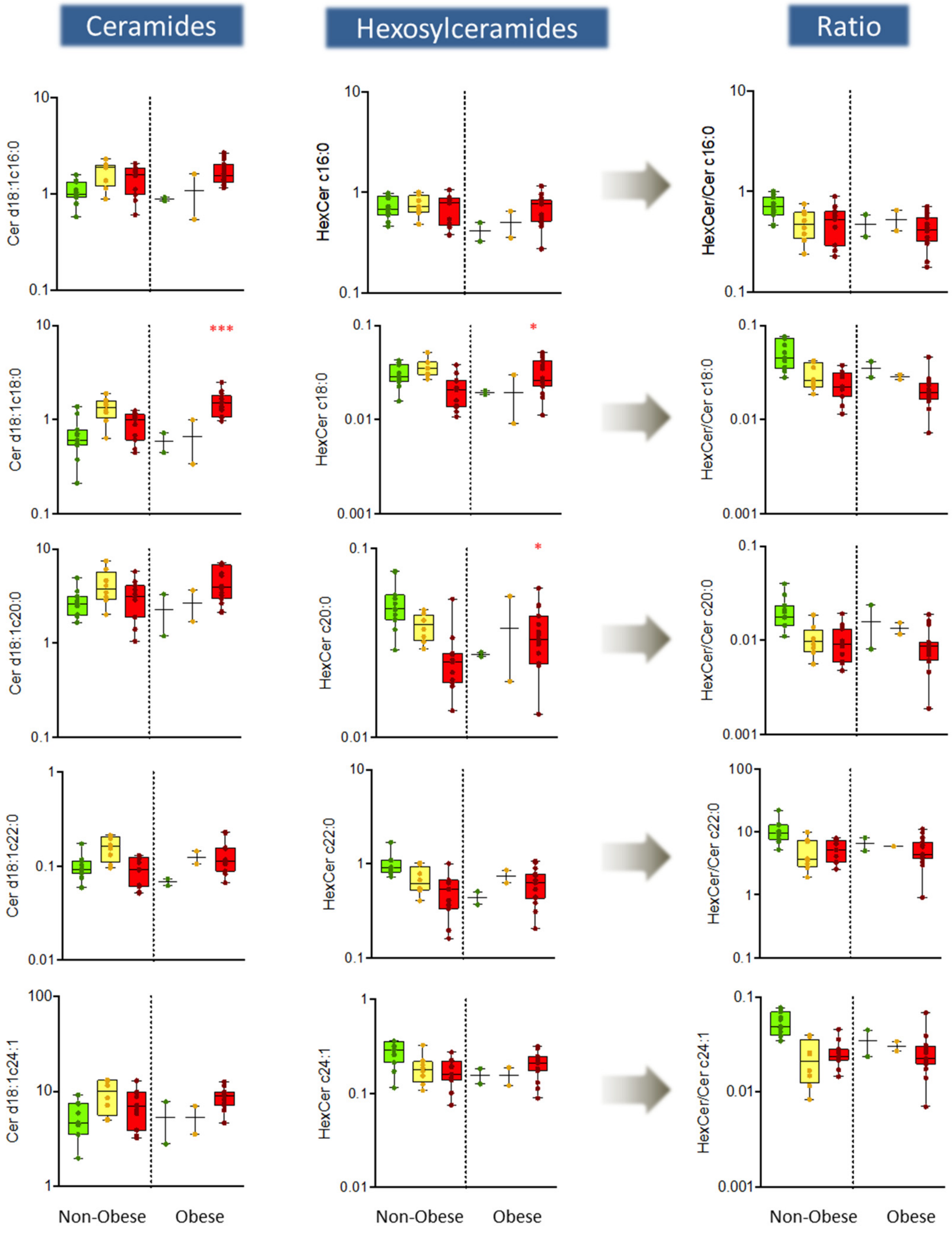

2.3. Figure S3. Plasma levels of metabolites included in tryptophan metabolism via the kynurenine pathway, in non-obese and obese patients in analysed groups.

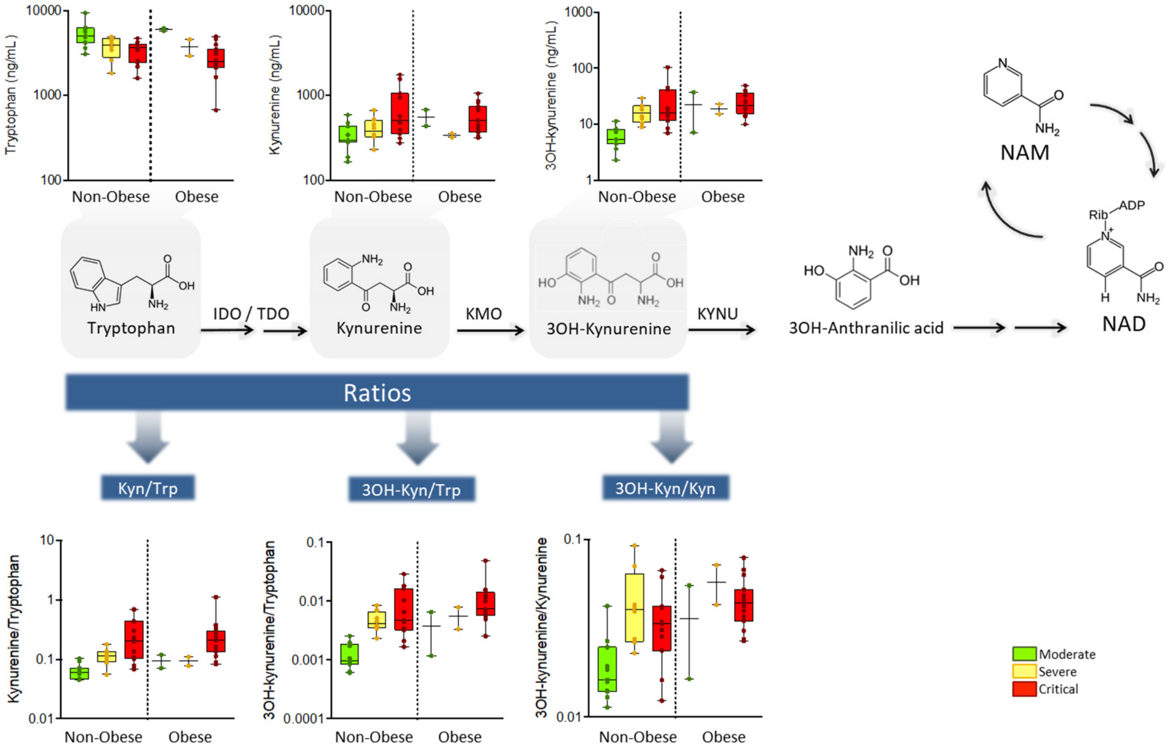

3. Supplementary Tables

3.1. Table S1. Characteristics of hospitalized COVID-19 patients included in the study by severity and obesity.

	Moderate n = 13		Severe n = 10		Critical n = 26	
	Non-Obese n = 11	Obese n = 2	Non-Obese n = 8	Obese n = 2	Non-Obese n = 11	Obese n = 15
**Demographics**						
Age, years	51 (16)	---	50 (15)	---	61 (11)	58 (10)
Gender, male	5 (45.5)	2 (100)	4 (50)	1 (50)	6 (54.5)	6 (40)
Body mass index (Kg/m^2)	23 (6)	---	26 (4)	---	25 (3)	36 (4) [a]
APACHE II score	6 (3)	---	11 (5)	---	18 (7)	15 (4)
**Chronic comorbidities**	3 (27.3)	1 (50)	5 (62.5)	1 (50)	9 (81.8)	9 (60)
Chronic lung disease	1 (9.1)	0 (0)	3 (37.5)	0 (0)	1 (9.1)	0 (0)
Asthma	0 (0)	0 (0)	1 (12.5)	0 (0)	0 (0)	0 (0)
COPD	1 (9.1)	0 (0)	1 (12.5)	0 (0)	1 (9.1)	0 (0)
Cardiovascular disease	2 (18.2)	1 (50)	3 (37.5)	0 (0)	7 (63.6)	8 (53.3)
Coronary artery disease	0 (0)	1 (50) [a]	0 (0)	0 (0)	0 (0)	0 (0)
Hypertension	2 (18.2)	1 (50)	3 (37.5)	0 (0)	7 (63.6)	8 (53.3)
Atrial fibrillation	0 (0)	0 (0)	0 (0)	0 (0)	0 (0)	1 (6.7)
Other medical conditions						
Immunosuppression	0 (0)	0 (0)	1 (12.5)	0 (0)	1 (9.1)	1 (6.7)
Alcoholism	2 (18.2)	1 (50)	1 (12.5)	0 (0)	1 (9.1)	2 (13.3)
Current or former smoker	2 (18.2)	0 (0)	1 (12.5)	0 (0)	3 (27.3)	3 (20)
Dislipidaemia	0 (0)	1 (50) [a]	1 (12.5)	1 (50)	1 (9.1)	7 (46.7) [a]
Diabetes mellitus	0 (0)	1 (50) [a]	2 (25)	0 (0)	3 (27.3)	4 (26.7)
Liver disease	1 (9.1)	0 (0)	0 (0)	0 (0)	0 (0)	0 (0)
Chronic renal failure	0 (0)	0 (0)	0 (0)	0 (0)	1 (9.1)	2 (13.3)
Haematological malignancies	1 (9.1)	0 (0)	0 (0)	0 (0)	0 (0)	0 (0)
Solid tumour	1 (9.1)	0 (0)	1 (12.5)	0 (0)	1 (9.1)	0 (0)
Hypothyroidism	1 (9.1)	0 (0)	0 (0)	0 (0)	0 (0)	2 (13.3)
HIV	1 (9.1)	0 (0)	0 (0)	0 (0)	0 (0)	0 (0)
**Chronic medications**						
Inhaled corticosteroids	0 (0)	0 (0)	0 (0)	0 (0)	1 (9.1)	0 (0)

Biological drugs	0 (0)	0 (0)	0 (0)	0 (0)	1 (9.1)	0 (0)
ACE inhibitors	0 (0)	1 (50) [a]	2 (25)	0 (0)	3 (27.3)	3 (20)
Angiotensin II blockers	1 (9.1)	0 (0)	0 (0)	0 (0)	1 (9.1)	2 (13.3)
Statins	0 (0)	1 (50) [a]	0 (0)	0 (0)	1 (9.1)	2 (13.3)
Oral corticosteroids	0 (0)	0 (0)	1 (12.5)	0 (0)	1 (9.1)	1 (6.7)
**Symptoms**						
Cough	6 (54.5)	2 (100)	8 (100)	1 (50) [a]	6 (54.5)	13 (86.7)
Fever	7 (63.6)	1 (50)	8 (100)	1 (50) [a]	10 (90.9)	13 (86.7)
Sore throat	1 (9.1)	0 (0)	0 (0)	0 (0)	0 (0)	0 (0)
Headache	3 (27.3)	1 (50)	1 (12.5)	2 (100) [a]	1 (9.1)	1 (6.7)
Rhinorrhea	0 (0)	0 (0)	1 (12.5)	0 (0)	0 (0)	0 (0)
Myalgia	3 (27.3)	1 (50)	2 (25)	2 (100)	1 (9.1)	1 (6.7)
Sputum	0 (0)	0 (0)	3 (37.5)	0 (0)	0 (0)	4 (26.7)
Diarrhea	2 (18.2)	1 (50)	1 (12.5)	1 (50)	1 (9.1)	4 (26.7)
Chest pain	1 (9.1)	1 (50)	1 (12.5)	0 (0)	1 (9.1)	2 (13.3)
Nausea/vomiting	1 (9.1)	0 (0)	1 (12.5)	0 (0)	0 (0)	1 (6.7)
Dyspnea	2 (18.2)	1 (50)	5 (62.5)	1 (50)	9 (81.8)	12 (80)
Altered mental status	0 (0)	0 (0)	0 (0)	0 (0)	1 (9.1)	1 (6.7)
Symptoms GAP	5 (3-7)	---	7 (4-12)	---	5 (3-8)	8 (6-11) [a]
**Treatment received in hospital before inclusion**						
Hydroxychloroquine	4 (36.4)	0 (0)	4 (50)	1 (50)	4 (36.4)	6 (40)
Systemic corticosteroids	0 (0)	0 (0)	4 (50)	1 (50)	2 (18.2)	4 (26.7)
Hydrocortisone	0 (0)	0 (0)	0 (0)	0 (0)	0 (0)	1 (6.7)
Methylprednisone	0 (0)	0 (0)	2 (25)	0 (0)	0 (0)	3 (20)
Dexamethasone	0 (0)	0 (0)	2 (25)	1 (50)	2 (18.2)	0 (0)
Tocilizumab	0 (0)	0 (0)	2 (25)	0 (0)	1 (9.1)	1 (6.7)
**Vital signs at inclussion**						
Temperature, ºC	37.1 (1.3)	---	35.8 (1.0)	---	35.8 (0.9)	36.1 (1.4)
Heart rate, bpm	82 (17)	---	81 (14)	---	74 (13)	80 (13)
Respiratory rate, rpm	22 (16-24)	---	26 (21-34)	---	30 (24-34)	24 (21-26)
Systolic blood pressure, mmHg	128 (17)	---	111 (18)	---	120 (22)	104 (22)
Diastolic blood pressure, mmHg	80 (11)	---	68 (13)	---	63 (15)	63 (17)
Mean blood pressure, mmHg	97 (11)	---	82 (13)	---	82 (16)	76 (17)

Oxygen saturation, %	98 (96-99)	---	98 (97-99)	---	94 (93-97)	94 (93-97)
Fraction of inspired oxygen, %	---	---	100 (82-100)	---	100 (60-100)	80 (55-100)
Arterial oxygen pressure, mmHg	---	---	91 (78-118)	---	89 (62-115)	77 (68-91)
P_aO_2/F_IO_2 ratio	---	---	104 (78-187)	---	107 (91-133)	110 (72-179)
S_aO_2/F_IO_2 ratio	---	---	98 (97-161)	---	98 (94-156)	116 (96-170)
**Analytical variables at inclusion**						
C-reactive protein, mg/mL	3.8 (4.1)	---	15 (11)	---	21.7 (14.0)	25 (12)
Procalcitonin, ng/mL	0.12 (0.03-0.12)	---	0.24 (0.08-0.64)	---	0.49 (0.27-1.48)	0.36 (0.16-1.13)
Leukocytes, $x10^3/\mu L$	6.3 (5.9-8.9)	---	7.36 (6.19-9.93)	---	8.27 (6.82-13.83)	9.31 (6.13-11.49)
Lymphocytes, $x10^3/\mu L$	1.5 (1.0-2.4)	---	0.87 (0.81-1.02)	---	0.68 (0.35-0.95)	0.87 (0.64-1.23)
IL-6, pg/mL	---	---	43.8 (4.3-102.0)	---	132.1 (22.8-266.2)	125.5 (37.4-205.5)
Fibrinogen, mg/dL	497 (390-500)	---	500 (398-500)	---	500 (500-500)	500 (500-500)
D-dimer, ng/mL	620 (377-780)	---	2250 (1022-3957)	---	1710 (1090-3980)	1610 (970-2560)
Lactate, mg/dL	1.13 (0.82-1.29)	---	1.62 (1.03-1.87)	---	1.75 (1.26-1.85)	1.42 (1.15-2.89)
LDH, U/L	212 (171-269)	---	424 (400-466)	---	541 (356-835)	413 (349-542)
Ferritin, µg/L	---	---	1009 (664-2040)	---	717 (673-2638)	965 (464-3790)
**Radiological findings at inclusión**						
Bilateral infiltrates	5 (45.5)	2 (100)	8 (100)	2 (100)	11 (100)	15 (100)
Ground glass	7 (63.6)	2 (100)	6 (75)	1 (50)	5 (45.5)	8 (53.3)
Consolidation	7 (63.6)	2 (100)	6 (75)	2 (100)	8 (72.7)	9 (60)

Data expressed as frequencies and percentages [n (%)] or mean (SD) or median (IQR)].

[a] $p \leq 0.05$ compared to non-obese.

3.2. Table S2. Sphingolipids and tryptophan metabolites in moderate, severe, and critical hospitalized COVID-19 patients.

	Moderate n = 13	Severe n = 10	Critical n = 26	p-value [d]
**Sphingolipids (resp.)**				
**Ceramides (Cer)**				
Cer C14:0	0.206 (0.082)	0.219 (0.107)	0.180 (0.069)	0.336
Cer C16:0 [a]	1.03 (0.27)	1.56 (0.56) [b]	1.59 (0.49) [b]	0.002
Cer C18:0 [a]	0.675 (0.308)	1.188 (0.466) [b]	1.251 (0.481) [b]	0.001
Cer C20:0	2.64 (0.97)	3.89 (1.77)	3.85 (1.77)	0.067
Cer C22:0 [a]	0.094 (0.029)	0.152 (0.044) [b]	0.113 (0.045) [c]	0.007
Cer C24:0 [a]	1.77 (0.45)	2.32 (0.71)	1.66 (0.72) [c]	0.036
Cer C24:1 [a]	5.43 (2.24)	8.70 (3.72) [b]	7.99 (2.64) [b]	0.010
**Hexosylceramides (HexCer)**				
HexCer C16:0	0.672 (0.204)	0.700 (0.199)	0.691 (0.222)	0.947
HexCer C18:0	0.029 (0.009)	0.033 (0.011)	0.027 (0.012)	0.273
HexCer C20:0 [a]	0.047 (0.016)	0.039 (0.010)	0.031 (0.012) [b]	0.002
HexCer C22:0 [a]	0.893 (0.315)	0.699 (0.209)	0.577 (0.253) [b]	0.004
HexCer C24:0 [a]	0.178 (0.109)	0.108 (0.068)	0.091 (0.042) [b]	0.003
HexCer C24:1 [a]	0.256 (0.088)	0.180 (0.064) [b]	0.191 (0.063) [b]	0.018
**Ratio HexCer/Cer**				
C16:0 [a]	0.669 (0.191)	0.492 (0.161)	0.466 (0.177) [b]	0.004
C18:0 [a]	0.048 (0.017)	0.029 (0.008) [b]	0.022 (0.008) [b]	0.000
C20:0 [a]	0.020 (0.008)	0.011 (0.004) [b]	0.009 (0.004) [b]	0.000
C22:0 [a]	10.01 (4.47)	5.15 (2.55) [b]	5.48 (2.39) [b]	0.000
C24:0 [a]	0.101 (0.054)	0.051 (0.037) [b]	0.061 (0.026) [b]	0.005
C24:1 [a]	0.051 (0.016)	0.024 (0.011) [b]	0.026 (0.012) [b]	0.000
**Tryptophan metabolism**				
**Metabolites** (ng/mL)				
Tryptophan [a]	5444 (1595)	3747 (1032) [b]	3024 (1085) [b]	0.000
Serotonin	19.2 (17.8)	14.9 (11.0)	11.3 (8.5)	0.326
5-Hydroxyindoleacetic acid	1.81 (1.94)	1.73 (1.54)	3.44 (3.42)	0.115

Kynurenine [a]	377 (150)	397 (126)	644 (381) [b]	0.016
Kynurenic acid	9.6 (7.45)	8.86 (2.25)	20.96 (26.3)	0.327
3OHKyn [a]	8.8 (8.9)	17.5 (6.3) [b]	26.1 (20.0) [b]	0.008
Anthranilic acid	0.083 (0.050)	0.070 (0.063)	0.126 (0.085)	0.080
Nicotinic acid	14.1 (8.1)	33.3 (23.5)	23.1 (20.7)	0.067
Nicotinamide	51.9 (38.2)	73.0 (66.8)	45.4 (34.9)	0.260
**Ratios**				
Kynurenine/tryptophan [a]	0.070 (0.022)	0.111 (0.034)	0.258 (0.224) [b]	0.003
Kynurenic acid/kynurenine	0.025 (0.009)	0.023 (0.005)	0.028 (0.021)	0.734
3OHKyn/kynurenine [a]	0.022 (0.013)	0.048 (0.023) [b]	0.041 (0.016) [b]	0.001
3OHKyn/tryptophan [a]	0.002 (0.002)	0.005 (0.002) [b]	0.011 (0.010) [b]	0.003
Serotonin/kynurenine [a]	0.060 (0.052)	0.044 (0.044)	0.022 (0.020) [b]	0.011
5-hydroxyindoleacetic/3OHKyn	0.218 (0.161)	0.103 (0.097)	0.140 (0.101)	0.120
**NAD consuming reactions**				
**Ratios**				
Cortisone/Cortisol [a]	0.120 (0.061)	0.062 (0.013) [b]	0.053 (0.024) [b]	0.000
Succinate/α-ketoglutarate	0.240 (0.073)	0.216 (0.073)	0.180 (0.114) [b]	0.203
Lactate/Pyruvate [a]	39.4 (34.8)	30.2 (10.3)	20.8 (7.3) [b, c]	0.021

[a] p≤0.05 between moderate, severe and critical in univariable ANOVA analysis.

[b] p≤0.05 compared to moderate

[c] p≤0.05 compared to severe

[d] p-value obtained by multivariable linear regression model adjusting by age and gender

Values are means (SD). Abbreviations: Cer: ceramides; HexCer: hexosylceramides; resp.: response; 3OHKyn: 3-hydroxykynurenine.

3.3. Table S3. Detailed list of 221 biomarkers studied in COVID-19 patients grouped according to their corresponding pathway.

**Analytes**		**Ratios**	
Neurotransmitters and polar compounds			
Creatinine	Glutamic acid	Tryptophan / long neutral amino acids	Glutamine / glutamic acid
Creatine	Acetyl carnitine	Tyrosine / long neutral amino acids	Tyrosine / phenylalanine
Valine	Carnitine	Phenylalanine / long neutral amino acids	Tryptophan / phenylalanine
Leucine	Phenylalanine	Leucine / long neutral amino acids	Acetylcarnitine / carnitine
Isoleucine	Tyrosine	Isoleucine / long neutral amino acids	Creatinine / creatine
Choline	Trimethylamine *N*-oxide	Valine / long neutral amino acids	Trimethylamine N-oxide / choline
Glutamine		Methionine / long neutral amino acids	Valine / tryptophan
Methionine		Aromatic amino acids / brain chained amino acids	
Tryptophan metabolism			
Tryptophan	Anthranilic acid	Kynurenine / tryptophan	Anthranilic acid / kynurenine
Serotonin	Nicotinic acid	Kynurenic acid / kynurenine	Anthranilic acid / tryptophan
5-Hydroxyindoleacetic acid	Nicotinamide	3-hydroxykynurenine / kynurenine	5-Hydroxyindoleacetic / acid tryptophan
Kynurenine		3-hydroxykynurenine / tryptophan	Nicotinic acid / nicotinamide
Kynurenic acid		Serotonin / kynurenine	N-acetylmethionine / 3-hydroxynynurenine
3-Hydroxykynurenine		5-hydroxyindoleacetic / 3-hydroxykynurenine	N-acetylmethionine / tryptophan
Carboxylic acids			
Lactate	α-ketoisocaproate	Citrate / pyruvate	Malate / glyoxylate
Pyruvate	β-Hydroxybutyrate	Isocitrate / citrate	Hydroxymethylbutyrate / α-ketoglutarate
Citrate	2-Hydroxyglutarate	α-ketoglutarate / isocitrate	α-ketoisocaproate / leucine
Isocitrate	Isovalerate	Succinate / α-ketoglutarate	Isovalerate / leucine
α-ketoglutarate		Fumarate / succinate	Hydroxymethylbutyrate / leucine
Succinate		Malate / fumarate	Acetoacetate / leucine
Fumarate		Lactate / pyruvate	β-hydroxybutyrate / leucine
Malate		Citrate / malate	Isovalerate / β-hydroxybutyrate
Hippurate		Acetoacetate / β-hydroxybutyrate	β-hydroxybutyrate / Branched-chain amino acids
Glyoxylate		2-Hydroxyglutarate / α-ketoglutarate	Acetoacetate / brached-chain amino acids
Hydroxymethylbutyrate		α-ketoglutarate / glutamate	Isovalerate / 3-hydroxykynurenine
Acetoacetate		Glyoxylate / isocitrate	2-Hydroxyglutarate / isocitrate
Lipids			
Diacylglycerol 16:1	Lysophosphatidylcholine 18:0	Ceramide d18:1 C22:0 / ceramide d18:1 C18:0	

Diacylglycerol 16:0	Ceramide d18:1 C14:0	Ceramide d18:1 C24:0 / ceramide d18:1 C16:0	
Diacylglycerol 16:0 18:2	Ceramide d18:1 C16:0	Monoacylglycerol 16:0 / diacylglycerol 16:0	
Diacylglycerol 18:1 16:0	Ceramide d18:1 C18:0	Monoacylglycerol 20:4 / diacylglycerol 20:4	
Diacylglycerol 16:0 18:0	Ceramide d18:1 C20:0	Monoacylglycerol 18:1 / diacylglycerol 18:1	
Diacylglycerol 18:2	Ceramide d18:1 C22:0	Monoacylglycerol 18:2 / diacylglycerol 18:2	
Diacylglycerol 18:0 18:2	Ceramide d18:1 C24:0	Hexosylceramide C16:0 / ceramide C16:0	
Diacylglycerol 18:1	Ceramide d18:1 C24:1	Hexosylceramide C18:0 / ceramide C18:0	
Diacylglycerol 18:0 18:1	Hexosylceramide C16:0	Hexosylceramide C20:0 / ceramide C20:0	
Diacylglycerol 18:0	Hexosylceramide C18:0	Hexosylceramide C22:0 ceramide C22:0	
Diacylglycerol 18:0 20:4	Hexosylceramide C20:0	Hexosylceramide C24:0 / ceramide C24:0	
Monoacyglycerol 16:0	Hexosylceramide C22:0	Hexosylceramide C24:1 / ceramide C24:1	
Monoacyglycerol 18:2	Hexosylceramide C24:0	Hexosylceramide C22:0 / hexosylceramide C18:0	
Monoacyglycerol 18:1	Hexosylceramide C24:1	Hexosylceramide C22:0 / ceramide C18:0	
Monoacyglycerol 20:4	Sphingosine		
Lysophosphatidylcholine 16:0			
Steroids			
Cortisol	Androstandiol-glucuronide	20α-dihydrocortisol / cortisol	Deoxycorticosterone / corticosterone
20α-dihydrocortisol	5β-Androstan-3α,17α-diol-17-glucuronide	20β-dihydrocortisol / cortisol	Cortisol / 11-deoxycortisol
20β-dihydrocortisol	Pregnandiol-glucuronide	20α-dihydrocortisol / 20β-dihydrocortisol	11-deoxycortisol / 11-hydroxyprogesterone
5α-tetrahydrocortisol	5-Androsten-3β,17β-diol-disulfate	5α-tetrahydrocortisol / cortisol	Testosterone / androstenedione
5β-tetrahydrocortisol	5-Androsten-3β,17α-diol-disulfate	5β-tetrahydrocortisol / cortisol	Etiocholanolone / testosterone
6β-hydroxycortisol	Androstenedione-3β,17β-disulfate	6β-hydroxycortisol / cortisol	Etiocholanolone glucuronide / androsterone glucuronide
Cortisone	5α,3α,17β-Adiol-3,17 bis(sulfate)	Cortisol / cortisone	Etiocholanolone sulfate / androsterone sulfate
20α-dihydrocortisone	5-Pregnen-3β,20α-diol bis(sulfate)	Cortisol metabolites / cortisone metabolites	Testosterone sulfate / testosterone
20β-dihydrocortisone	5α-Pregnan-3β,20α-diol bis(sulfate)	20α-dihydrocortisone / cortisone	Dehydroepiandrosterone sulfate / androstenedione
5α-tetrahydrocortisone	21-hydroxypregnenolone bis(sulfate)	20β-dihydrocortisone cortisone	5β-Androstan-3α,17β-diol-17-sulfate / dehydroepiandrosterone sulfate
5β-tetrahydrocortisone	5β-Pregnandiol-3-sulfate-20α-glucuronide	20α-dihydrocortisone / 20β-dihydrocortisone	5α-androstan-3α,17β-diol-17-sulfate / dehydroepiandrosterone sulfate
Corticosterone	Dehydroepiandrosterone-sulfate	5α-tetrahydrocortisone cortisone	5α-androstan-3β,17β-diol-17-sulfate / Dehydroepiandrosterone sulfate
11-dehydrocorticosterone	Epiandrosterone-sulfate	5β-tetrahydrocortisone cortisone	Estradiol sulfate / estrone sulfate
Deoxycorticosterone	Etiocholanolone-sulfate	11-dehydrocorticosterone / corticosterone	estradiol sulfate / testosterone
11-deoxycortisol	5β-Androstan-3β,17β-diol-3-sulfate	11-dehydrocorticosterone / cortisone	estradiol sulfate / testosterone sulfate
17-hydroxyprogesterone	5α-Androstan-3α,17β-diol-3-sulfate		
Etiocholanolone	5α-Androstan-3β,17β-diol-3-sulfate		
Androstenedione	16α-hydroxy-dehydroepiandrosterone-3-sulfate		

Testosterone	Pregnenolone-sulfate	
Testosterone sulfate	Pregnanolone-sulfate	
Epiandrosterone-sulfate	5α-Pregan-3β,20α-diol-3-sulfate	
Androsterone-sulfate	5β-Pregan-3β,20α-diol-3-sulfate	
5β-Androstan-3α,17β-diol-17-sulfate	5α-Pregan-3β,20α-diol-20-sulfate	
5α-Androstan-3α,17β-diol-17-sulfate	5-Pregnenolone-3-sulfate	
5α-Androstan-3β,17β-diol-17-sulfate	Tetrahydrocortisone glucuronide	
Androsterone-glucuronide	Estrone sulfate	
Etiocholanolone-glucuronide	Estradiol sulfate	
Tetrahydrocortisol glucuronide		

3.4. Table S4. Standards and reagents, and their corresponding suppliers

Pathway	Standards and reagents	Supplier
Tryptophan metabolism	Tryptophan (Trp)	SA
	Serotonin (5HT)	SA
	5-Hydroxyindoleacetic acid (5HIAA)	SA
	Kynurenine (Kyn)	SA
	Kynurenic acid (KA)	SA
	3-Hydroxy kynurenine (3OHKyn)	SA
	Phenylalanine	SA
	Tyrosine	SA
	Tyrosine	SA
	Valine	SA
	Leucine	SA
	Isoleucine	SA
	Methionine	SA
	Tryptophan-d_5	Alsachim
	Kynurenine-$^{13}C_6$	Alsachim
	3-Hydroxy kynurenine-$^{13}C_6$	Alsachim
	5-Hydroxyindoleacetic acid-d_4	Alsachim
	Kynurenine acid-d_5	TRC
	Serotonin-d_5	TRC
Carboxylic acids	Lactic acid (LA)	SA
	Pyruvic acid (PyA)	SA
	Citric acid (CA)	SA
	Isocitric acid (IA)	SA
	Succinic acid (SA)	SA
	Fumaric acid (FA)	SA
	Malic acid (MA)	SA
	Hippuric acid	SA
	Tryptophan	SA
	Lactic acid-$^{13}C_3$	SA
	Pyruvic acid-$^{13}C_3$	TRC
	Citric acid-d_4	SA
	Succinic acid-d_4	SA
	Fumaric acid-$^{13}C_4$	SA
	Malic acid-d_3	CIL
	Tryptophan-d_5	TRC
	N-(3-Dimethylaminopropyl)-N'-ethylcarbodiimide hydrochloride	SA
	o-benzyl hydroxylamine	SA
	Hydrochloric acid	Merck
	Pyridine	Merck
Lipids	Diacylglycerol 16:0-d_5	APL
	Diacylglycerol 16:1-d_5	APL
	Diacylglycerol 18:0-d_5	APL
	Diacylglycerol 18:1-d_5	APL
	Diacylglycerol 18:2-d_5	APL

	Diacylglycerol 20:4-d_5	APL
	Ceramide 18:1 C16:0-d_7	APL
	Ceramide 18:1 C18:0-d_7	APL
	Ceramide 18:1 C24:0-d_7	APL
	Ceramide 18:1 C24:1-d_7	APL
Neurotransmitters	Creatinine	SA
	Creatine	SA
	Valine	SA
	Leucine	SA
	Isoleucine	SA
	Choline	SA
	Glutamine	SA
	Glutamic acid	SA
	Acetyl carnitine	SA
	Carnitine	SA
	Phenylalanine	SA
	Tryptophan	SA
	Tyrosine	SA
	Trimethylamine N-oxide	SA
	Creatinine-d_3	TRC
	Glutamine-d_5	TRC
	Glutamic acid-^{13}C-^{15}N	TRC
	Phenylalanine-d_5	TRC
	Tryptophan-d_5	TRC
	Tyrosine-d_4	TRC
	Trimethylamine N-oxide-d_9	SA
Steroids	Cortisol (F)	Steraloids
	20α-dihydrocortisol (20aDHF)	Steraloids
	20β-dihydrocortisol (20bDHF)	Steraloids
	6β-hydroxycortisol (6OHF)	Steraloids
	Cortisone (E)	Merck
	20α-dihydrocortisone (20aDHE)	Steraloids
	20β-dihydrocortisone (20bDHE)	Steraloids
	6β-hydroxycortisone (6OHE)	Steraloids
	Corticosterone (B)	Steraloids
	11-dehydrocorticosterone (A)	Steraloids
	Testosterone (T)	Steraloids
	Epitestosterone (epiT)	Steraloids
	Androstenedione (AED)	Steraloids
	Progesterone (Prog)	Steraloids
	Epiandrosterone-sulfate (epiANS)	Steraloids
	Androsterone-sulfate (ANS)	Steraloids
	Androsterone-glucuronide	LGC
	Etiocholanolone-glucuronide	LGC
	5β,3α,17α-Adiol-17-glucuronide	LGC
	Pregnandiol-glucuronide	Steraloids
	Androstenedione-3β,17β-disulfate	Lab synthesis
	5α,3α,17β-Adiol-3,17 bis(sulfate)	Lab synthesis

	5α,3β,17β-Adiol-3,17 bis(sulfate)	Lab synthesis
	5β,3β,20α-Pregnandiol bis(sulfate)	Lab synthesis
	5α,3β,20α-Pregnandiol bis(sulfate)	Steraloids
	3β,21-Dihydroxypregn-5-en-20-one bis(sulfate)	Lab synthesis
	5β-Pregnandiol-3-sulfate-20α-glucuronide	Lab synthesis
	Dehydroepiandrosterone-sulfate (DHEAS)	Steraloids
	Epiandrosterone-sulfate (epiANS)	Steraloids
	Etiocholanolone-sulfate (EtioS)	Steraloids
	5β,3α,17β-Adiol-3-sulfate	Lab synthesis
	5α,3α,17β-Adiol-3-sulfate	Lab synthesis
	5α,3β,17β-Adiol-3-sulfate	Lab synthesis
	16α-hydroxy-dehydroepiandrosterone-3-sulfate	Steraloids
	Pregnanolone-sulfate	Steraloids
	Pregnanolone-sulfate	Steraloids
	5α,3β,20β-Pregnandiol-3-sulfate	Steraloids
	5β-Pregnandiol-20-one-3-sulfate	Steraloids
	Estradiol-sulfate (E2S)	Steraloids
	6β-hydroxycortisol-d_4	TRC
	Cortisol-d_4	SA
	Testosterone-d_3	NMI
	Epitestosterone-d_3	LGC
	Epitestosterone-d_3-sulfate	LGC
	Epitestosterone-d_4-glucuronide	LGC
	Epiandrosterone-$^{18}O_3$-sulfate	Lab synthesis
	Androsterone-d_4-glucuronide	NMI
	Estradiol-d_3-sulfate	LGC
	5α,3α,17β-Adiol-3,17-disulfate-$^{18}O_3$	Lab synthesis
	5α,3β,17β-Adiol-3,17-disulfate-$^{18}O_3$	Lab synthesis
	5α,3β,17β-Adiol-3-sulfate-$^{18}O_3$-17-glucuronide	Lab synthesis
Common material	Water milliQ	MI
	Ammonium formate	SA
	Methanol	Merck
	Formic acid	Merck
	Acetonitrile	Merck
	Ethyl Acetate	Merck

4. References

1. Gomez-Gomez A, Soldevila A, Pizarro N, Andreu-Fernandez V, Pozo OJ. Improving liquid chromatography-tandem mass spectrometry determination of polycarboxylic acids in human urine by chemical derivatization. Comparison of o-benzyl hydroxylamine and 2-picolyl amine. *Journal of pharmaceutical and biomedical analysis* 2019; **164**: 382-94.

2. Olesti E, Rodríguez-Morató J, Gomez-Gomez A, Ramaekers JG, de la Torre R, Pozo OJ. Quantification of endogenous neurotransmitters and related compounds by liquid chromatography coupled to tandem mass spectrometry. *Talanta* 2019; **192**: 93-102.

3. Velázquez AM, Roglans N, Bentanachs R, et al. Effects of a Low Dose of Caffeine Alone or as Part of a Green Coffee Extract, in a Rat Dietary Model of Lean Non-Alcoholic Fatty Liver Disease without Inflammation. Nutrients 2020; 12(11).

4. Marcos J, Renau N, Valverde O, et al. Targeting tryptophan and tyrosine metabolism by liquid chromatography tandem mass spectrometry. *Journal of chromatography A* 2016; **1434**: 91-101.

5. Gomez-Gomez A, Miranda J, Feixas G, et al. Determination of the steroid profile in alternative matrices by liquid chromatography tandem mass spectrometry. *The Journal of steroid biochemistry and molecular biology* 2020; **197**: 105520.

6. Pozo OJ, Marcos J, Khymenets O, et al. SULFATION PATHWAYS: Alternate steroid sulfation pathways targeted by LC-MS/MS analysis of disulfates: application to prenatal diagnosis of steroid synthesis disorders. *Journal of Molecular Endocrinology* 2018; **61**(2): M1-m12.
